# Revealing Detailed Cartilage Function Through Nanoparticle Diffusion Imaging: A Computed Tomography & Finite Element Study

**DOI:** 10.1007/s10439-024-03552-7

**Published:** 2024-07-16

**Authors:** Juuso Tuppurainen, Petri Paakkari, Jiri Jäntti, Mikko T. Nissinen, Maria C. Fugazzola, René van Weeren, Sampo Ylisiurua, Miika T. Nieminen, Heikki Kröger, Brian D. Snyder, Anisha Joenathan, Mark W. Grinstaff, Hanna Matikka, Rami K. Korhonen, Janne T. A. Mäkelä

**Affiliations:** 1 Department of Technical Physics, University of Eastern Finland, POB 1627, 70211 Kuopio, Finland; 2 Diagnostic Imaging Center, Kuopio University Hospital, Kuopio, Finland; 3 Department of Clinical Sciences, Faculty of Veterinary Medicine, Utrecht University, Utrecht, The Netherlands; 4 Research Unit of Health Sciences and Technology, University of Oulu, Oulu, Finland; 5 Department of Diagnostic Radiology, Oulu University Hospital, Oulu, Finland; 6 Department of Orthopaedics and Traumatology, Kuopio University Hospital, Kuopio, Finland; 7 Kuopio Musculoskeletal Research Unit, University of Eastern Finland, Kuopio, Finland; 8 Department of Orthopedic Surgery, Boston Children’s Hospital, Boston, USA; 9 Departments of Biomedical Engineering and Chemistry, Boston University, Boston, USA

**Keywords:** Computational modeling, Constituent-specific behavior, Contrast-enhanced computed tomography, Dual-contrast agent, Photon-counting detector, Osteoarthritis

## Abstract

The ability of articular cartilage to withstand significant mechanical stresses during activities, such as walking or running, relies on its distinctive structure. Integrating detailed tissue properties into subject-specific biomechanical models is challenging due to the complexity of analyzing these characteristics. This limitation compromises the accuracy of models in replicating cartilage function and impacts predictive capabilities. To address this, methods revealing cartilage function at the constituent-specific level are essential. In this study, we demonstrated that computational modeling derived individual constituent-specific biomechanical properties could be predicted by a novel nanoparticle contrast-enhanced computer tomography (CECT) method. We imaged articular cartilage samples collected from the equine stifle joint (*n* = 60) using contrast-enhanced micro-computed tomography (µCECT) to determine contrast agents’ intake within the samples, and compared those to cartilage functional properties, derived from a fibril-reinforced poroelastic finite element model. Two distinct imaging techniques were investigated: conventional energy-integrating µCECT employing a cationic tantalum oxide nanoparticle (Ta_2_O_5_-cNP) contrast agent and novel photon-counting µCECT utilizing a dual-contrast agent, comprising Ta_2_O_5_-cNP and neutral iodixanol. The results demonstrate the capacity to evaluate fibrillar and non-fibrillar functionality of cartilage, along with permeability-affected fluid flow in cartilage. This finding indicates the feasibility of incorporating these specific functional properties into biomechanical computational models, holding potential for personalized approaches to cartilage diagnostics and treatment.

## Introduction

Articular cartilage has the very challenging task of withstanding significant mechanical stresses during daily activities such as walking or running. Consequently, cartilage possesses a distinctive composition and structure that endow it with the necessary biomechanical properties. Articular cartilage is primarily composed of proteoglycans (PGs), collagens, and fluid, which all play a crucial role in its biomechanical behavior [32]. The collagen network and fluid pressure allow cartilage to withstand impacts and cyclic loading, whereas PGs with their capability to bind water define the equilibrium stiffness of the tissue when the free fluid has flown out during a prolonged loading [9–11, 21].

The biomechanics of cartilage are critical for joint function and, therefore, profound understanding of it is essential. To deepen our understanding, computational models are used to simulate scenarios inside and outside of the experimental range, enabling investigations into the individual constituents’ (collagen and proteoglycan) impact on cartilage mechanical behavior, stresses, strains, and fluid flow [10, 52]. Computational models have increasingly gained popularity since the early 2000s, and consistently, they have been demonstrated to be effective in mimicking cartilage behavior [31, 35, 61]. One such advanced model is the fibril-reinforced poroelastic (FRPE) material model, which has been applied and validated several times, showing its ability to capture the complex behavior and distinguish between normal and osteoarthritic cartilage [9–11, 26, 27, 31, 41, 52]. The model enables us to analyze cartilage function at a constituent-specific level, meaning that we can extract functional properties that describe proteoglycan-defined behavior (non-fibrillar matrix modulus *E*_nf_), collagen-defined behavior (both initial *E*_f_^0^ and strain-dependent fibril network modulus *E*_f_^*ε*^), and fluid flow (permeability *k*_0_ and permeability strain-dependency factor *M*). The non-fibrillar matrix modulus *E*_nf_ determines tissue equilibrium stiffness and resilience, with higher values indicating greater resistance to prolonged deformation (e.g., standing). The initial fibril network modulus *E*_f_^0^ represents collagen stiffness at the onset of deformation (e.g., jumping), while the strain-dependent modulus *E*_f_^*ε*^ captures collagen’s nonlinear behavior and stiffening under strain. Permeability *k*_0_ and its strain-dependency *M* regulate fluid flow, impacting load bearing, and hydration. Higher permeability, which reflects increased porosity, promotes faster tissue relaxation, while lower permeability results in higher fluid pressurization. These aspects are challenging to explore through conventional analytical biomechanical measurements. This is particularly significant because fluid flow, which is determined by the collective of solid constituents, is recognized as a key contributor to cartilage health and function [39, 40].

For the knee joint model generation and simulation, various medical imaging methods, e.g., magnetic resonance imaging, standard radiography, and computed tomography (CT), are utilized [15, 46, 49, 55, 63]. However, providing data on individual cartilage constituent-specific biomechanical properties (material properties) is exceedingly difficult [56]. Instead, these imaging techniques provide cartilage volume and topography information but not data on the constituents which is critical to gaining a comprehensive understanding of the intricacies of cartilage mechanics [17, 56].

To develop more accurate computational models and to assess the condition of the cartilage, the use of advanced imaging techniques such as contrast-enhanced computed tomography (CECT) is highly advantageous. Contrast agents can be designed to selectively target specific cartilage constituents, such as PGs and collagen, or reflect porosity [1, 4, 13, 58]. Furthermore, CECT capability improves when combined with photon-counting detectors (PCD-CT) [54]. Compared to conventional energy-integrating detectors (EIDs), PCDs detect individual photons and classify them by their energy, enabling spectral imaging and the separation of multiple contrast agents with a single scan. Importantly, this is achieved without increasing radiation doses or the need for cumbersome co-registering of multiple image sets. To harness this feature, we have reported a quantitative dual-energy CT technique [3–5, 18–20, 54, 58] that combines an ‘active’ cationic contrast agent and a ‘passive’ neutral contrast agent. The cationic contrast agent binds to anionic PGs [33, 45, 57, 58], while neutral contrast agent reflects free water content and steric hindrance created by the solid constituents of the cartilage [54, 58]. Moreover, the dual-contrast method can be further refined by converting measured concentrations to relative partitions (i.e., dividing the measured concentration by the original concentration), and unifying them (i.e., dividing the active contrast agent partition by the partition of the neutral agent) to form combined partition. This approach merges free water, proteoglycans, and steric hindrance effects, thereby enhancing the sensitivity of CECT imaging [3, 5, 19, 58]. Numerous prior studies [1, 2, 30, 33, 45, 50, 57, 58] have already showcased the capacity of CECT to capture cartilage constituents, structures, and biomechanics defined from analytical experiments. However, an unexplored aspect is the integration of contrast agents' diffusion data directly into the material parameters of the finite element (FE) cartilage models. Such an approach offers a fresh and intricate perspective on the relationship between contrast agent diffusion and cartilage function, potentially offering refinement for subject-specific models. By providing biomechanical parameters of the target tissue, such as permeability, this methodology could significantly enhance the precision of the advanced cartilage models [47, 48, 53] and ultimately lead to more accurate simulation outcomes.

The primary aim of this study is to quantify cartilage function through contrast-enhanced micro-computed tomography (µCECT) imaging utilizing computational modeling. To achieve this, FRPE FE modeling of cartilage function under experimental compression is conducted to determine the constituent-specific material properties. Contrast agent diffusion experiments are performed using conventional EID-µCT and experimental PCD-µCT setups to follow the uptake of cationic and neutral contrast agents. We hypothesize that cartilage permeability, extracted from FE modeling, serves as a highly sensitive biomarker for µCECT imaging parameters to assess cartilage function. This hypothesis builds upon previous studies that have demonstrated the ability of cationic contrast agents to target negatively charged PGs [33, 45, 57, 58] and the neutral contrast agent’s dependence on porous structure [54, 58], which together define the permeability. The secondary aim is to evaluate the efficacy of the dual-contrast agent and the PCD-µCT method in assessing FE model derived cartilage function. To accomplish this, we will use both single- and dual-contrast agents and employ EID- and PCD-based µCT setups, respectively.

## Materials and Methods

### Samples

The samples used in this study were previously utilized in a prior publication [16]. Thirty equine stifle joints were carefully chosen (joints with macroscopic abnormalities were excluded), and cylindrical osteochondral plugs of 10 mm depth and 8.5 mm diameter were harvested using a hollow drill. Samples were harvested from two different sites within the joint to account for variations in biomechanics and structures: the distal intertrochlear groove (*n* = 30) and the medial femoral condyle (*n* = 30). Separate single-contrast and dual-contrast experiments were performed on batches of fifteen samples from each group (*n* = 30). Cartilage thickness measurements were obtained using µCT (Nikon XT H 225, Nikon Metrology Europe, Leuven, Belgium) by measuring the samples in the air without any presence of contrast agents. Samples were inside a sealed container during the imaging to prevent drying.

### Biomechanics

**Fig. 1:**
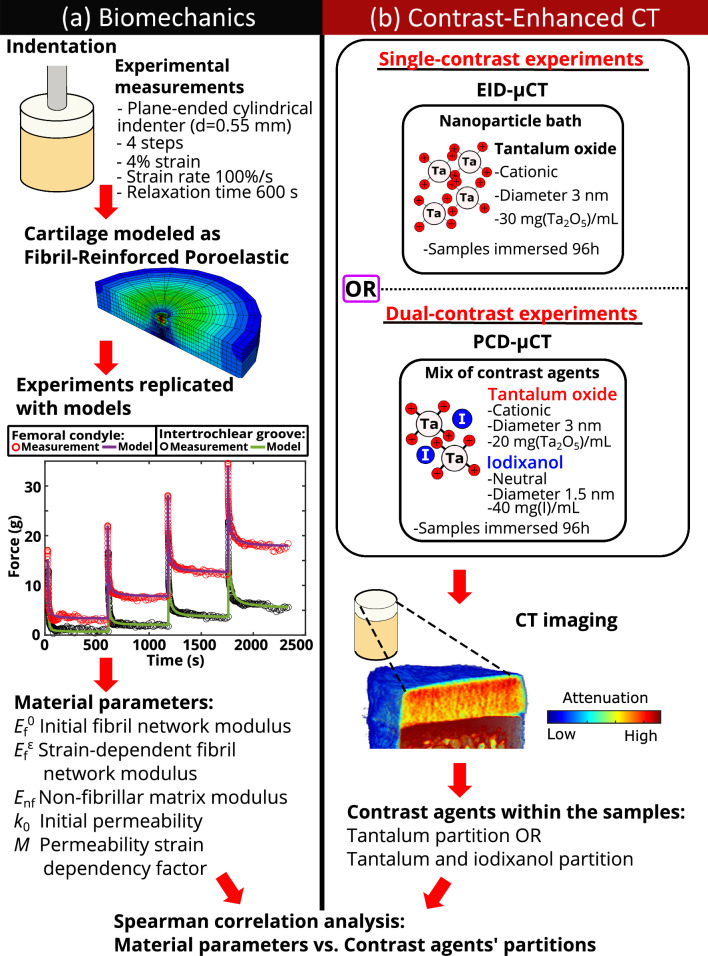
The study workflow: **a** Indentation testing (*n* = 60) using a stress-relaxation protocol was followed by modeling samples as Fibril-Reinforced Poroelastic (FRPE) materials, optimizing five material parameters. **b** Half of the samples (*n* = 30) immersed in a Ta_2_O_5_-cNP bath were imaged using EID-µCT, while the other half (*n* = 30) immersed in a Ta_2_O_5_-cNP and iodixanol mixture were imaged using PCD-µCT. Contrast agent partitions within samples were calculated from the images based on attenuation. Material parameters and contrast agent partitions were then statistically compared using Spearman correlation.

The experimental tests were conducted in indentation geometry (Fig. 1a). The measuring geometry consisted of a displacement actuator with a resolution of 0.1 µm (PM1A11939, Newport, Irvine, CA, USA) and a load cell of 0.25 kg (Model 31, Honeywell International Inc., Charlotte, NC, USA). A goniometer was applied to ensure perpendicularity between the cartilage surface and the plane-ended cylindrical indenter. The indenter diameter was 0.55 mm. The samples were fixed with three screws through the subchondral bone. The sample holder was then filled with phosphate-buffered saline (PBS, 310 mOsmol/L).

Full contact on surfaces between the indenter and the cartilage was ensured with an equilibrium pre-stress of 40 kPa, resulting in minor deformation (from 8 to 60 µm). Cartilage recovery time was set to 600 s after preliminary tests, ensuring equilibrium before each indentation test. The testing protocol included four steps of compression, which corresponded to 4% of the remaining cartilage thickness. The ramp rate was 100% per second relative to cartilage thickness, and each compression step was followed by 600 s of relaxation time.

### Computational Modeling

A sample-specific axisymmetric FRPE FE model was created to replicate experimental stress-relaxation tests (Fig. 1a) using Abaqus (v6.12-3, Dassault Systèmes, Vèlizy-Villacoublay, France). The total stress tensor is defined as the sum of stress tensors caused by the fibrillar and non-fibrillar matrices, excluding (pore) fluid pressure [9, 10]:

1 $${\sigma }_{\text {t}}={\sigma }_{\text {nf}}+{\sigma }_{\text {f}}-pI,$$

where ${\sigma }_{\text {nf}}$ and ${\sigma }_{\text {f}}$ are the stress tensors of the non-fibrillar and fibrillar matrices, $p$ is the fluid pressure, and $I$ is the unit tensor. The stress tensor of the Neo-Hookean hyperelastic non-fibrillar matrix is [10, 52]:

2 $${\sigma }_{\text {nf}}=\frac{1}{2}{K}_{\text {nf}}\frac{\left(J-1\right)}{J}I+\frac{1}{J}{G}_{\text {nf}}\left(F{F}^{\text{T}}-{J}^\frac{2}{3}I\right),$$

where ${\sigma }_{\text {nf}}$ is the stress tensor of the non-fibrillar matrix, ${K}_{\text {nf}}$ and ${G}_{\text {nf}}$ are the bulk and shear moduli of the non-fibrillar matrix, respectively, $F$ is the deformation gradient tensor, $J$ is the determinant of the $F$ and $I$ is the unit tensor. The bulk and shear moduli of the non-fibrillar matrix can be expressed as a function of the elastic modulus (*E*_nf_) and Poisson’s ratio (${v}_{\text {nf}}$ = 0.42 [24, 25, 31]) of the non-fibrillar matrix [10, 40]:

3 $${K}_{\text {nf}}=\frac{{E}_{\text {nf}}}{3\left(1-2{v}_{\text {nf}}\right)},$$

4 $${G}_{\text {nf}}=\frac{{E}_{\text {nf}}}{2\left(1+{v}_{\text {nf}}\right)}.$$

Darcy’s law is employed to describe the fluid flow inside the porous matrix as follows:

5 $$q=-k\nabla p,$$

where $q$ is the rate of the fluid flow, $k$ is the permeability of the material, and $\nabla p$ is the pressure gradient. The deformation in the porous material causes a change in the void ratio and, hence, the permeability changes, which is then described as follows [60]:

6 $$k={k}_{\text {0}}{\left(\frac{1+e}{1+{e}_{\text {0}}}\right)}^{M},$$

where $k$ *and* ${k}_{\text {0}}$ are the current and initial values of the permeability, $e$ and ${e}_{\text {0}}$ are the current and the initial values for the void ratio, respectively, and *M* is a constant describing the deformation dependency of permeability. The value for the initial void ratio was set to 3.5 [25, 35, 36], yielding a 78% fluid volume fraction, which corresponds to the average of earlier reported fluid volume fraction throughout the cartilage depth [35, 38]. The collagen fibril network is modeled as a nonlinear elastic fibrillar matrix [10, 52]. The stress in tension of an individual collagen fibril was modeled as follows [10, 37, 52]:

7 $${\sigma }_{\text {f}, i}=\frac{1}{2}{E}_{\text {f}}^{\varepsilon }{\varepsilon }_{\text {f}, i}^{2}+{E}_{\text {f}}^{\text {0}}{\varepsilon }_{\text {f}, i},$$

where ${\sigma }_{\text {f}, i}$ and ${\varepsilon }_{\text {f}, i}$ are stress and strain of the *i*:th fibril, *E*_f_^0^ is the initial fibril network modulus, and *E*_f_^*ε*^ is the strain-dependent fibril network modulus. The collagen network was created using primary and secondary fibrils, and their ratio was defined using a relative density. The relative density describes how much more of the stress is applied to the primary fibrils [61]:

8 $${\sigma }_{\text {f,p}}={\rho }_{\text {z}}C{\sigma }_{\text {f}},$$

and

9 $${\sigma }_{\text {f,s}}={\rho }_{\text {z}}{\sigma }_{\text {f}},$$

where ${\sigma }_{\text {f,p}}$ is primary fibril stress, ${\sigma }_{\text {f,s}}$ is secondary fibril stress, $C$ is the relative density and ${\rho }_{\text {z}}$ is a factor accounting for fibril density as a function of depth, which is equal to 1 in our model. The collagen network consisted of four organized primary fibrils and thirteen randomly oriented secondary fibrils, with a relative density of 12.16 between the primary and secondary fibrils [7, 26, 40, 61, 62]. The primary fibrils’ direction and number were determined in a way that they form a mesh-like structure parallel to the surface. It was achieved with four fibrils at every node. The secondary fibrils were employed in the *x*-, *y*-, and *z*-directions, and in all directions at 45° angles to the *x*-, *y*-, and *z*-axes, totaling 13 secondary fibrils as originally suggested by Wilson et al. [61].

FE mesh convergence was ensured and the meshes consisted of 350 linear axisymmetric pore pressure continuum elements. To replicate the experimental tests, five sample-specific model parameters were optimized: strain-dependent fibril network modulus (*E*_f_^*ε*^), non-fibrillar matrix modulus (*E*_nf_), initial permeability (*k*_0_), and permeability strain-dependency factor (*M*). Each optimized model parameter contributes uniquely to the biomechanical output, meaning that different combinations of moduli and permeability cannot produce identical behavior, particularly when considering multiple strain-dependent stress-relaxation steps [8, 52]. The optimization was done by minimizing the mean-squared relative error between the measured and simulated reaction forces in the 2nd, 3rd, and 4th stress-relaxation steps [9, 40, 52]. The axisymmetric boundary conditions and limitations to displacements were applied similarly like in previous studies [9, 40, 52]. The structure, composition, and fluid fraction/void ratio of each sample were assumed to be homogenous in order to obtain mechanical properties independent from the composition and structure of the tissue, as done previously [10, 31, 41, 52].

### µCECT Imaging

Before µCECT experiments, samples were cut into quarters. To allow the contrast agent diffusion only through the articulating surface, the sides of the quarter samples utilized in µCECT experiments were sealed with glue (ethyl cyanoacrylate) before immersing them in a contrast agent bath. µCECT experiments were conducted using both single- and dual-contrast techniques (Fig. 1b). In single-contrast experiments, one contrast agent was utilized, and the samples were imaged with an EID-µCT device. In turn, dual-contrast experiments were conducted using the mix of two contrast agents, and the samples were imaged with a PCD-µCT setup.

### Single-Contrast Experiments (Utilizing EID-µCT)

A quarter of each sample was immersed in a bath containing cationic tantalum oxide nanoparticle contrast agent (Ta_2_O_5_-cNP, hydrodynamic diameter = 2.55 ± 0.96 nm, concentration 30 mg(Ta_2_O_5_)/mL [14, 33]) for 96 h and imaged in air with EID-µCT, i.e., a conventional µCT device (Nikon XT H 225, voxel size 40 × 40 × 40 µm). The X-ray spectrum was generated with 150 kVp voltage, 0.17 mA current, and filtered with a 0.5 mm copper filter [23]. Flat-field calibration was utilized to calibrate the detector.

### Dual-Contrast Experiments (Utilizing PCD-µCT)

A quarter from each sample was utilized for dual-contrast experiments by immersing it for 96 hours in a bath containing a mix of cationic Ta_2_O_5_-cNP (hydrodynamic radius 2.55 ± 0.96 nm) and neutral iodixanol (approximated spherical size ~ 1.5 nm). The concentrations of the contrast agents in the bath were 20 mg(Ta_2_O_5_)/mL and 40 mg(I)/mL for Ta_2_O_5_-cNP and iodixanol, respectively. A custom-built PCD-µCT setup (detector: XCounter XC-Flite FX15, Danderyd, Sweden, voxel size 68 × 68 × 68 µm) was utilized, and the X-ray source (VJX IXS1203 Mini-Focus, Bohemia, NY, USA) was configured with a voltage of 120 kVp, a current of 0.25 mA, and filtered with 3.0 mm aluminum and 0.5 mm copper filters [54]. To enable spectral imaging, the lower and upper thresholds were set to 10 keV and 80 keV, respectively, giving us three distinctive energy bins: low (10–80 keV), high (80–120 keV), and total (10–120 keV) energy bins. For the dual-contrast analysis, only low and total energy bins were used. Signal-to-thickness calibration was utilized to calibrate the detector [28, 29, 54].

### Image Analysis

The uptake of contrast agent was analyzed with a custom-made MATLAB (R2020b, MathWorks, Natick, MA, USA) script. The script was used to determine contrast agent partitions within the samples by subtracting bulk cartilage attenuation (Hounsfield unit) at the 0-h timepoint from the attenuation at the 96-h timepoint and dividing the result by the initial contrast agent bath attenuation [18, 54].

For dual-contrast experiments, a calibration-based material decomposition was utilized to accurately separate the two contrast agent concentrations, as previously described [3, 18, 54]. Similarly, the material decomposition was validated using multiple mixtures containing various ratios of both contrast agents. The estimated contrast agent concentrations were converted to partitions correspondingly. Combined tantalum-iodine partition was calculated by dividing Ta_2_O_5_-cNP partition by the iodixanol partition.

### Statistical Analysis

Linear correlation analysis (Spearman) was used to determine the relationships between the optimized model parameters and contrast agent partitions. The Mann–Whitney *U*-test was used to compare the differences in contrast agents’ partitions and material model parameters between methods and the two locations. The threshold for statistical significance was set at *p* < 0.05. Statistical analysis was conducted using MATLAB.

## Results

The FE models accurately replicated the experimental stress-relaxation data (*R*^2^ = 0.95 ± 0.05, Fig. 1a). The mean values and 95% confidence intervals (CIs) of the optimized FE model parameters are shown in Table 1. A statistically significant difference in material parameters was found only in the non-fibrillar matrix modulus *E*_nf_ and initial permeability *k*_0_ between locations. The non-fibrillar matrix modulus *E*_nf_ was higher in the medial femoral condyle compared to the distal intertrochlear groove (*p* < 0.001).

**Table 1:** Mean values (95% confidence interval) of the sample-specific FE model parameters.

µCECT method	Location	*E*_f_^0^ (MPa)	*E*_f_^*ε*^ (MPa)	*E*_nf_ (MPa)	*k*_0_ (m^4^/Ns)* 10^−15^	*M*
Single	Distal intertrochlear groove	0.29 (0.21–0.37)	10.1 (5.2–15.0)	0.13*** (0.09–0.16)	4.7 (3.7–5.7)	20.5 (17.2–23.7)
Single	Medial femoral condyle	0.31 (0.21–0.41)	7.3 (4.8–9.7)	0.42*** (0.34–0.50)	3.3 (2.4–4.2)	21.6 (16.1–27.3)
Dual	Distal intertrochlear groove	0.28 (0.21–0.36)	7.2 (4.5–9.9)	0.10*** (0.06–0.13)	5.1*** (4.1–6.1)	20.1 (15.3–24.6)
Dual	Medial femoral condyle	0.25 (0.16–0.34)	8.6 (6.6–10.6)	0.42*** (0.32–0.52)	3.2*** (2.6–3.8)	24.2 (20.2–28.2)

*E*_f_^0^ is the initial fibril network modulus, *E*_f_^*ε*^ is the strain-dependent fibril network modulus, *E*_nf_ is the non-fibrillar matrix modulus, *k*_0_ is the initial permeability, *M* is the permeability strain-dependency factor, and ‘µCECT method’ indicates which µCECT acquisition method was used for the respective sample group after biomechanical measurements. Asterisks (*) indicate statistically significant differences (*p < 0.05, **p < 0.01, ***p < 0.001) in the FE model parameters between distal intertrochlear groove and medial femoral condyle

For the µCECT measurements, the mean bulk partitions and 95% CIs are shown in Table 2. The Ta_2_O_5_-cNP diffusion was elevated without the presence of iodixanol (*p* < 0.001). Furthermore, there was a notably higher Ta_2_O_5_-cNP partition in the medial femoral condyle compared to the distal intertrochlear groove (*p* < 0.001). Conversely, the partition of iodixanol was increased in the distal intertrochlear groove compared to the medial femoral condyle (*p* < 0.01).

**Table 2:** Mean contrast agent partition (95% confidence interval) of bulk partitions within the samples and calculated combined partition (AU = arbitrary unit)

Location	µCECT method	Ta_2_O_5_-cNP	Iodixanol	Combined (AU)
Distal intertrochlear groove	Single	313.7%^†††,▲▲▲^ (282.0–345.4)		
	Dual	177.9%^†††,▲▲^ (150.9–204.9)	58.0%^▲▲▲^ (56.2–59.8)	3.1^▲▲▲^ (2.6–3.5)
Medial femoral condyle	Single	704.4%^†††,▲▲▲^ (649.8–758.9)		
	Dual	270.0%^†††,▲▲^ (223.2–316.7)	51.6%^▲▲▲^ (48.8–54.4)	5.3^▲▲▲^ (4.4–6.2)

Symbols indicate significant differences in contrast agents’ partitions between single- and dual-contrast agent methods (^†^) and two locations (^▲^). ^†,▲^*p* < 0.05, ^††,▲▲^*p* < 0.01,^†††, ▲▲▲^*p* < 0.001

**Fig. 2:**
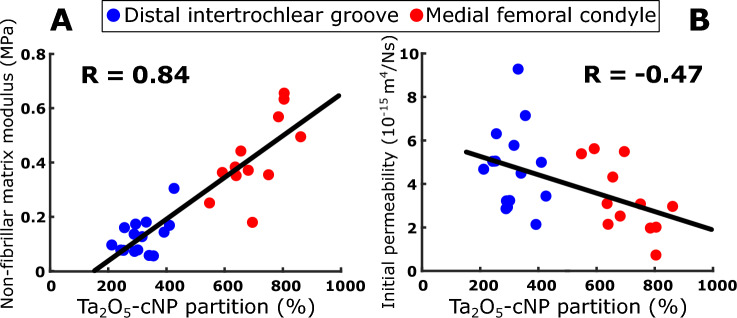
The observed single-contrast experiment Spearman correlation of Ta_2_O_5_-cNP with non-fibrillar matrix modulus *E*_nf_ (**A**) and initial permeability *k*_0_ (**B**).

**Fig. 3:**
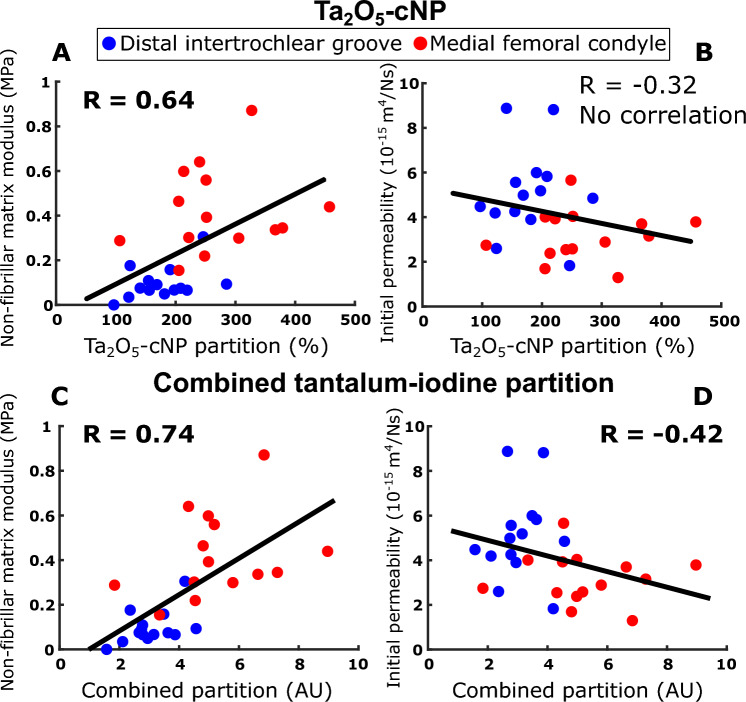
The observed dual-contrast experiment Spearman correlation of Ta_2_O_5_-cNP. The correlation of Ta_2_O_5_-cNP partition with non-fibrillar matrix modulus *E*_nf_ (**A**) and initial permeability *k*_0_ (**B**) is enhanced by combining it with iodixanol partition (**C** and **D**, respectively).

**Fig. 4:**
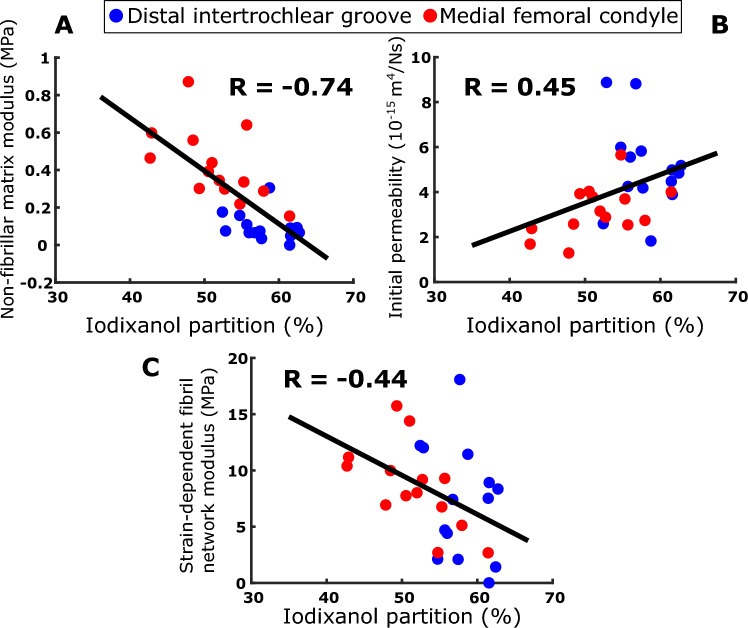
The observed dual-contrast experiment Spearman correlation of iodixanol partition with non-fibrillar matrix modulus *E*_nf_ (**A**), initial permeability *k*_0_ (**B**) and strain-dependent fibril network modulus *E*_f_^*ε*^ (**C**).

The single-contrast Ta_2_O_5_-cNP partition correlated positively and negatively with the non-fibrillar matrix modulus *E*_nf_ and the initial permeability *k*_0_, respectively (*R* = 0.84 and *R* = − 0.47, respectively, Fig. 2A, B). The dual-contrast combined tantalum-iodine partition correlated positively and negatively with the fibrillar matrix modulus *E*_nf_ and the initial permeability *k*_0_, respectively (*R* = 0.74 and *R* = − 0.42, respectively, Fig. 3C, D). The iodixanol partition negatively correlated with the non-fibrillar matrix modulus *E*_nf_ and the strain-dependent fibril network modulus *E*_f_^*ε*^, and positively correlated with the initial permeability *k*_0_: *R* = − 0.44, *R* = − 0.74, *R* = 0.45 (Fig. 4A–C)*.* All correlations between contrast agents’ partitions and model parameters are shown in the figures, and no additional statistically significant correlations (*p* < 0.05) were observed.

## Discussion

In this study, we investigated the ability of CT contrast agents to reveal FE model derived cartilage function, by using single- and dual-contrast methods. For the single-contrast measurements, we used Ta_2_O_5_-cNPs, while in dual-contrast measurements, we used a mixture of Ta_2_O_5_-cNPs and iodixanol. The two contrast agents are strikingly different in composition, size, and charge, and as such their diffusion patterns within cartilage vary [30, 33, 34, 45, 57, 58]. The diffusion of the larger and positively charged Ta_2_O_5_-cNPs depends on the negatively charged PGs [22, 33, 45, 57, 58], while the diffusion of the smaller and neutral iodixanol molecule reflects the amount of free water and porosity [54, 58]. Based on the results, Ta_2_O_5_-cNPs and iodixanol allow revealing specific functional properties of cartilage, including permeability as we hypothesized, the non-fibrillar matrix modulus *E*_nf_, and the strain-dependent fibril network modulus *E*_f_^*ε*^.

When examining data collected from the single-contrast experiments, the strongest correlation was between Ta_2_O_5_-cNP partition and non-fibrillar matrix modulus *E*_nf_ (*R* = 0.84, Fig. 2A). This correlation can be attributed to the presence of negatively charged PGs, to which the cationic nanoparticle is attracted [22, 33, 45, 57, 58]. PGs serve as a primary constituent influencing the non-fibrillar matrix modulus *E*_nf_, as highlighted in prior research [10, 11, 31, 41]. Thus, the increased Ta_2_O_5_-cNP diffusion correlates with the higher stiffness of the non-fibrillar structure of the target tissue.

The observation of a negative correlation between Ta_2_O_5_-cNP partition and initial permeability *k*_0_ was in contrast with that typically seen for conventional small molecule contrast agents where a positive correlation exists (Fig. 2B) [6, 43, 44, 51]. This unexpected finding (particularly in the context of larger charged nanoparticles like Ta_2_O_5_-cNP), where the influence of the charge supersedes the size-related effects, challenges the conventional understanding [6, 44, 51]. Typically, a higher proteoglycan (PG) content associates with lower permeability, indicative of better overall tissue health [59]. However, our results introduced a new and more nuanced perspective, suggesting that electrostatic attraction overrides the anticipated limiting effect of steric hindrance on diffusion. This conclusion is supported by the robust positive correlation observed between Ta_2_O_5_-cNP and the non-fibrillar matrix modulus *E*_nf_, coupled with its simultaneous negative correlation with permeability. Thus, the observed complex interplay among charge, matrix properties, and permeability aligns with a higher PG content contributing to healthier tissue, leading to lower permeability. These findings provide critical insights into the determination of overall cartilage health, particularly when assessed with a single-contrast agent.

Previous research has shown that the combined use of cationic and neutral contrast agents enhanced correlations with cartilage constituent properties and allowed the correlations to be reached at earlier time points [3, 5, 19, 58]. In our dual-contrast experiments, we observed higher correlation coefficients with the combined tantalum-iodine partition (Fig. 3A, B) compared to the Ta_2_O_5_-cNP partition alone in dual-contrast experiments (Fig. 3C, D). It is noteworthy that the results obtained with Ta_2_O_5_-cNP partition in single-contrast experiments, conducted with a separate set of samples, were consistent with the results obtained with combined partition in dual-contrast experiments (Figs. 2, 3). The combined tantalum-iodine partition correlated positively with the non-fibril matrix modulus *E*_nf_ and negatively with initial permeability *k*_0_. In addition, it is worth noting that the Ta_2_O_5_-cNP partition inside the samples decreased in dual-contrast experiments compared to single-contrast experiments (Table 2). This reduction in Ta_2_O_5_-cNP intake was most likely because the smaller iodixanol diffused more rapidly into the cartilage, simultaneously reducing the diffusion of Ta_2_O_5_-cNP. Importantly, this dynamic did not compromise the correlations, as the inclusion of iodixanol is advantageous for enhancing the characterization of cartilage.

The diffusion of neutral contrast agents, like iodixanol, is mainly influenced by free water and steric hindrance [54, 58]. Consequently, iodixanol partition positively correlated with initial permeability *k*_0_ in the present study (Fig. 4B), as increased permeability can be considered to boost iodixanol intake. The inverse correlation with the non-fibrillar matrix modulus *E*_nf_ can be attributed to a secondary correlation, as PGs influence tissue porosity [6, 12, 44], affirming that iodixanol diffusion is not dependent on the electrostatic attraction of PGs. The diffusion of iodixanol also negatively correlated with the strain-dependent fibril network modulus *E*_f_^*ε*^. This parameter characterizes the strain-dependent behavior of the collagen fibril network, and it is known to depend more on the organization or the architecture of the collagen network, than on collagen quantity [11]. Thus, this result indicates that the tightly organized collagen structure prevents iodixanol from entering the cartilage, which further suggests the potential utility of iodixanol as a reflector of the organized structure of collagen. Previous literature supports this finding, by showing the influence of collagen on both strain-dependent fibril network modulus *E*_f_^*ε*^ [10] and the diffusion of neutral nanoparticles [4].

In summary, dual-contrast enables the characterization of more functional parameters, compared to a single-contrast agent (Figs. 2, 3, 4). The contrasting correlations observed between Ta_2_O_5_-cNP and iodixanol with cartilage functional properties (Figs. 2A, B & 4A, B) underscore the intricate interplay between contrast agent behavior and cartilage composition and structure. These findings emphasize the significance of selecting the appropriate contrast agent when assessing cartilage function through µCECT methods. The choice of contrast agent yields valuable insights into specific facets of cartilage function. Furthermore, PCD technology provides a platform for spectral imaging, which is essential for multi-contrast methods, and removes the need for image co-registration making the analysis less laborious and faster. In the light of these findings and regarding the secondary aim of this study to evaluate the effectiveness of dual-contrast in conjunction with PCD-µCT, it is evident that this method demonstrates effectiveness in cartilage functional characterization.

In terms of limitations, this study employed equine samples. Although the characteristics of equine articular cartilage are known to be very close to those of human cartilage [42], this still could pose a limitation if we aim to extend our research findings to humans. The behavior of contrast agents in human cartilage may not necessarily mirror that in equine cartilage. Additionally, it is important to note that this study was conducted in an ex vivo setting using osteochondral plugs, and the transferability of these findings to whole joints, especially in vivo, requires further investigation. Emphasizing that in vivo studies face challenges, and obtaining permits for the use of contrast agents under development is a complex and time-consuming process. Alternatively, considering the promising results obtained with the contrast agents used in this study, future investigations may explore the in vivo applicability of the methodology using just the clinically approved iodixanol. Finally, four samples were excluded from the study because we could not replicate their mechanical response with our model. Nonetheless, the model proved suitable for all other samples, justifying its utilization. Also, one sample was excluded due to cracks in the articulating surface originating from sample drilling. Considering the total number of samples (*n* = 60) in the study, excluding those five samples had minimal impact on our results.

In conclusion, by utilizing FE modeling, which has not been done before in this context, we gain a more detailed evaluation of cartilage function compared to analytically derived equilibrium and instantaneous moduli [1, 3, 12, 33]. Computational modeling allows for the separation of function between specific structural components, whereas parameters determined solely through analytical methods may be more influenced by interactions among multiple structural components (e.g., Ebrahimi et al. [10]). Furthermore, these models enable a more nuanced estimation of cartilage properties arising from the porosity and water flow, which are otherwise challenging to define experimentally. Given the significant impact of early-stage osteoarthritis on cartilage composition and properties, particularly permeability [40], exploring methods to image functional properties presents an exciting avenue for advancing our understanding of cartilage biomechanics. These techniques hold potential for the development of personalized computational models based on imaging data.

Our study demonstrates that single- and dual-contrast µCECT are capable of assessing the intricate functional properties of cartilage, derived from FE modeling. Specifically, the dual-contrast approach enables a thorough assessment of the functional condition of the cartilage at a constituent-specific level, the distinctive attributes of Ta_2_O_5_-cNP serving as an excellent reflector of the non-fibrillar matrix, and neutral iodixanol, unveiling permeability with sensitivity to fibrillar stiffness. The ability to independently monitor the partition of contrast agents, or combine their partition, further expands our results, providing a more nuanced understanding of cartilage dynamics. This comprehensive insight holds promise for integrating such detailed information into future cartilage models, for the accurate prediction of the onset of osteoarthritis, and for assessing the precise effects of treatment.
